# Interleukin-6 and C-Reactive Protein Are Overexpressed in the Liver of Perinatal Deaths Diagnosed with Fetal Inflammatory Response Syndrome

**DOI:** 10.1155/2014/252780

**Published:** 2014-02-10

**Authors:** Lívia Helena M. Pereira, Juliana R. Machado, Janaínna G. P. Olegário, Laura P. Rocha, Marcos V. Silva, Camila S. O. Guimarães, Marlene A. Reis, Lúcio Roberto Castellano, Fernando S. Ramalho, Rosana R. M. Corrêa

**Affiliations:** ^1^Biological Sciences Department, General Pathology Division, Triangulo Mineiro Federal University, 38025-180 Uberaba, MG, Brazil; ^2^Biological Sciences Department, General Immunology Division, Triangulo Mineiro Federal University, 38025-180 Uberaba, MG, Brazil; ^3^Human Immunology Research and Education Group, Technical Health School of UFPB, Federal University of Paraiba, 58051-900 João Pessoa, PB, Brazil; ^4^Pathology and Forensic Medicine Department, Ribeirão Preto Faculty of Medicine of São Paulo University, 14049-900 Ribeirão Preto, SP, Brazil

## Abstract

Anatomopathologic studies have failed to define the fetal inflammatory response syndrome (FIRS) as a cause of fetal death. Here, liver fragments of perinatal autopsies were collected at a university hospital from 1990 to 2009 and classified according to the cause of death, perinatal stress, and gestational age (GA) of the fetus. IL-6, TNF-**α**, and C-reactive protein (CRP) expression were immunostained, respectively, with primary antibody. Cases with congenital malformation, ascending infection, and perinatal anoxia showed increased IL-6, CRP, and TNF-**α**, respectively. Prematures presented higher expression of IL-6 whereas term births showed higher expression of CRP. Cases classified as acute stress presented higher expression of IL-6 and TNF-**α** and cases with chronic stress presented higher expression of CRP. GA correlated negatively with IL-6 and positively with CRP and TNF-**α**. Body weight correlated negatively with IL-6 and positively with CRP and TNF-**α**. Despite the diagnosis of FIRS being clinical and based on serum parameters, the findings in the current study allow the inference of FIRS diagnosis in the autopsied infants, based on an *in situ* liver analysis of these markers.

## 1. Introduction

Fetal inflammatory response syndrome (FIRS) is a systemic inflammatory response originally described as the elevation of IL-6 levels in the fetal plasma. It is frequently associated with preterm delivery, premature rupture of the membranes, funisitis, chorioamnionitis, and adverse perinatal consequences, such as neonatal morbidity and mortality [1].

During an established infection in the intrauterine space the endotoxins produced by microorganisms might favor the release of cytokines and proteins within fetal and maternal organs [2]. This stress condition activates the complement system in the fetus, leading to a fetal inflammatory response characterized by a synthesis of proinflammatory substances such as tumor necrosis factor-alpha (TNF-*α*), interleukin-6 (IL-6), IL-1*β* and IL-8. These cytokines may serve as good markers to FIRS and may act in the liver cells triggering the synthesis of acute-phase proteins such as procalcitonin (PCT) and the C-reactive protein (CRP) [3].

The fetal liver is one of the most important organs to be analyzed in a perinatal autopsy due to its metabolic functions during the fetal development [4, 5]. This organ is enormously affected by intrauterine stress episodes, and morphological changes in this organ, such as steatosis, fibrosis, and foci of extramedullary erythropoiesis, are associated with perinatal death [6]. Altered hepatocytes change the pattern of CRP production, which in turn may serve as another marker of FIRS [7]. The concomitant measurement of CRP and IL-6 in umbilical cord blood and the occurrence of funisitis might help define FIRS diagnosis [1, 8].

There is a lack of anatomopathologic studies which could define the FIRS and its diagnostic markers as a cause of fetal death. One study evaluating infants who went through different kinds of stresses, such as intrauterine anoxia, infections, and congenital malformations, demonstrated that these children presented higher birth weight than the expected for the GA as well as a series of alterations in different organs, including the liver [6]. Possibly, the expression of the markers of FIRS in the liver of autopsied children might explain the increased weight gain and these markers may undergo variations according to the cause of death.

The aim of this study was to map the expression of the main inflammatory cytokines and proteins involved in the diagnosis of FIRS in autopsied children, as their influence on fetal weight, cause of death, and perinatal stress.

## 2. Material and Methods

### 2.1. Samples

Liver fragments of 48 perinatal autopsies conducted from gestational week 22 to postnatal day 7, in the General Hospital of Triângulo Mineiro Federal University, Minas Gerais, Brazil, between 1990 and 2009, were recovered. Exclusion criteria were cases of perinatal autopsies with incomplete records and protocols; biopsy blocks and slides unavailable in the archives; and cases of autolysed liver samples.

Enrolled cases were matched for gestational age and cause of death, following, literature definition [9]. For the morphologic analysis, only causes of death such as congenital malformation (CM), perinatal anoxia (PA) before delivery and ascending infection (AI) were investigated (Table 1). Samples were classified according to the perinatal stress defined when the thymus, adrenal, and liver presented morphological alterations compatible with intrauterine stress. The adrenal presented increasing amounts of coarse lipid droplets in the fetal cortex. The thymus was evaluated according to the presence of phagocytosis (positive cells for CD68 antibody), cortex thickness, and the weight for involution. The amount of intrahepatic hematopoiesis was evaluated in the liver. The placenta was also examined in these cases in order to confirm the autopsy findings. The clinical data on these patients was not available because most of them did not have a prenatal follow-up. Control sample included children with central nervous system and congenital kidney malformations. Children with these types of malformations had no signs of suffering in the intrauterine environment, and died due to lack of efficiency of the central nervous system or due to pulmonary hypoplasia, in cases of renal malformations, after birth. In other causes of death, hypoxia, and infection, still within the intrauterine environment these fetuses suffered with a hostile environment and developed changes consistent with stress. Therefore, the acute stress was characterized by events occurring at or after birth that might have been the causative agent of fetal death. On the other hand, chronic stress was defined by the response to injuries of long duration beginning in the intrauterine period and remaining until birth was related to the pathogenesis of perinatal death [10, 11]. The cases were grouped as acute or chronic perinatal stress (Table 1).

Data about anthropometric measurements, weight, GA, and clinical complications were collected from the autopsy records. Gestational age was determined through hallux-calcaneus length, which is considered to be the most reliable method to determine gestational age in autopsies [12]. Children with GA less than 37 weeks were considered premature [13] and those with GA greater than 24 weeks and Apgar score zero in the first minute were considered stillborn [14]. The children were classified according to the ratio between weight and gestational age into small for gestational age-SGA (weight is below the 10th percentile); appropriate for gestational age-AGA (weight is between the 10th and 90th percentiles) and large for gestational age-LGA (weight is above the 90th percentile) [15]. Values of expected weight for population were based on previous reports [16].

### 2.2. Immunohistochemistry

Paraffin-embedded liver fragments were sectioned and immunostained for IL-6, TNF-*α*, and CRP detection. The immunostaining was performed in a single batch by using the primary anti-IL-6 (1 : 600 Abcam), anti-TNF-*α* (1 : 50 DBS), and anti-CRP (1 : 1000 Abcam) antibodies. Quantization of immunostained IL-6, TNF-*α* and CRP *in situ* was performed using conventional light microscope using Leica QWin Plus image analysis software (*Leica Microsystems*, Wetzlar, Germany). Cumulative average method was used to determine the number of measures [17], showing a pattern result of 67 measures per slide. The results were expressed in percentage of the immunostained area per field. The diagnosis of fetal inflammatory response syndrome was done after obtaining the inflammatory markers values on liver fragments of perinatal autopsies.

Statistical analysis was conducted with *SigmaStat 2.03* software (SPSS Inc., Chicago, IL, USA). In cases of normal distribution and similar variances, Student's *t*-test was used in the comparison of two groups. Otherwise, Mann-Whitney *U* test was used in the comparison between two groups and Kruskal-Wallis *H* test in the comparison between three or more groups. Correlation between the two variables with nonnormal distribution was analyzed by the Spearman correlation test (rS). Differences in which “*P*” was less than 5% (*P* < 0.05) were considered statistically significant.

This study was approved by the Triângulo Mineiro Federal University (UFTM) Research Ethics Committee, protocol number 1316.

## 3. Results

**Figure 1:**
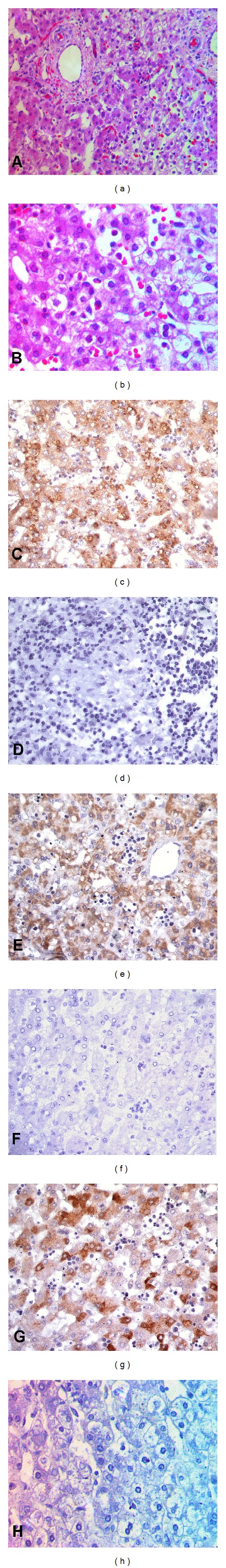
Liver fragments of perinatal autopsies: HE, 620x (a); HE, 1250x (b); IL-6 (c); IL-6 negative control (d); TNF-*α* (e); TNF-*α* negative control (f); PCR (g); PCR negative control (h) (anti-IL-6, anti- TNF-*α*, and anti-CRP, 620x).

Analysis of the expression of IL-6, TNF-*α*, and CRP in the liver of perinatal autopsies is presented in Table 2 and Figure 1.

**Figure 2:**
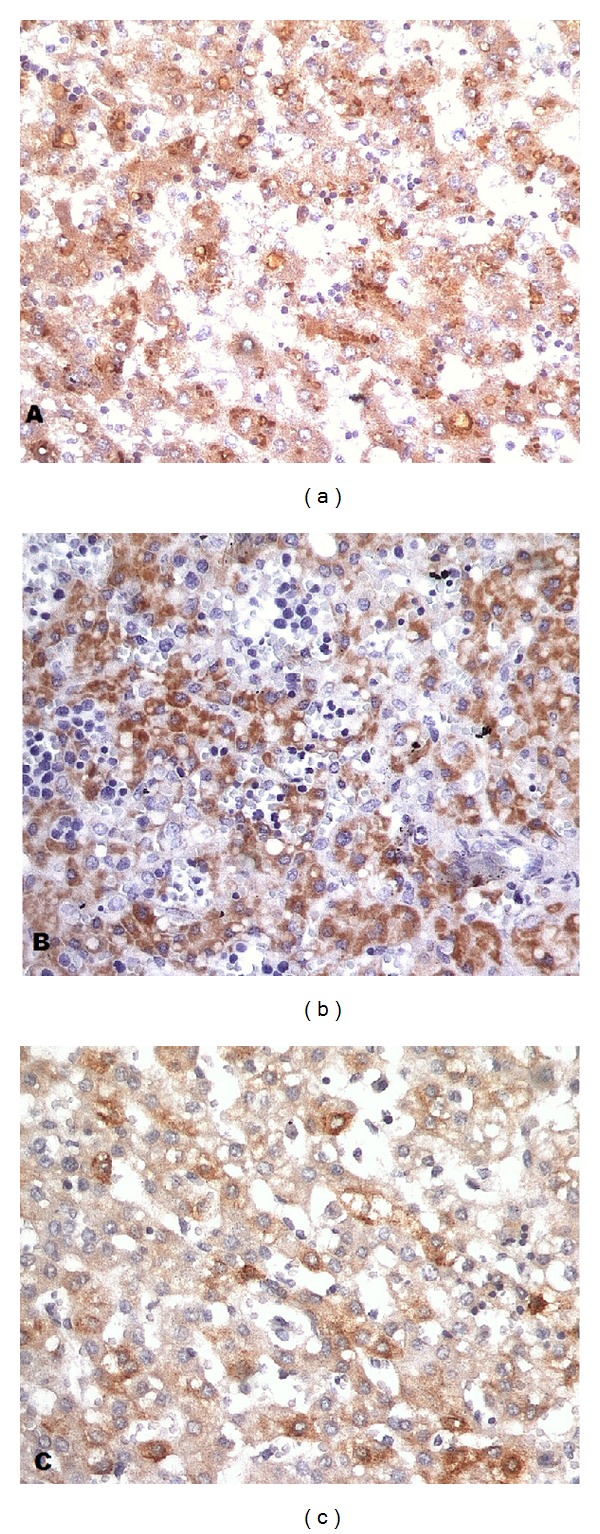
Cases with greater immunostaining for each antibody in liver fragments of perinatal autopsies: IL-6, case with congenital malformation (a); TNF-*α*, case with perinatal anoxia (b); and C-reactive protein (CRP), case with ascending infection (c) (anti-IL-6, anti-TNF-*α*, and anti-CRP, 620x).

Evaluation of IL-6 immunstaining revealed that its expression was significantly higher in cases with CM than in PA or AI (Figure 2) as well as in those cases classified as acute stress in comparison to chronic stress and also in samples from premature than term birth. Moreover, IL-6 expression correlated negatively with GA (rS = −0.314, *P* ≤ 0.001) and body weight (rS = −0.470, *P* ≤ 0.001). No differences were observed on IL-6 expression among SGA, LGA, and AGA groups.

The expression of CRP was significantly higher in cases with AI (Figure 2), chronic stress, and term births. In a separate assessment of preterm and term births, prematures with infection presented significantly lower expression of CRP. AGA newborns presented significant lower expression of CRP than the SGA and LGA groups. There was a positive and significant correlation between CRP and GA (rS = 0.379, *P* = 0.0315) and between the expression of CRP and body weight (rS = 0.290, *P* ≤ 0.001).

Cases with PA as well as cases with acute stress presented higher expression of TNF-*α* staining (Figure 2). The expression of TNF-*α* among term and preterm infants was not significant. The expression of TNF-*α* positively correlated with GA (rS = 0.222, *P* ≤ 0.001) and body weight (rS = 0.038, *P* = 0.032).

The weight of children with PA, AI, and CM was significantly higher than expected for the population (Table 3).

## 4. Discussion 

Fetuses with FIRS usually present elevated levels of proinflammatory cytokines detected in cord blood. Increased serum levels of IL-6, CRP, and TNF-*α* have been used as good markers of FIRS diagnosis. Recently, it has been shown that patients with FIRS present significant changes in the total white blood cells and a significant neutrophilia in cord blood samples [18]. It would suggest that these hematological parameters would be directly implicated in the changes observed in the proinflammatory cytokines present in blood samples. Moreover, all these information would indirectly implicate that other organs would exert important role in the inflammatory status observed in FIRS. Among all fetal organs, it was demonstrated that, in the lungs, the *in situ* expression of melatonin and cytokines seems to be related to the cause of death and type of stress [11]. Considering all organs, the liver seems to play an important role in the generation or maintenance of the inflammatory status observed in FIRS and that would be reflected in the cord blood samples. The liver enzymes function as immodulators being associated with the generation of substances implicated in the acute-phase response. The lack of definitive data motivated the search for the markers in liver samples of fetus diagnosed with FIRS.

Studies on neonates revealed that the peak of IL-6 production occurs 2-3 hours after exposition to a stressor agent and decreases after 6 to 8 hours [19]. In accordance with our data, cases with CM or acute stress underwent the action of the stressor agent only during delivery. Therefore, the increased expression of IL-6 found in this group may be related to this short time interval between stimulus and death. In the other causes of death, the IL-6 levels could be in the reduction phase. In our results, premature children presented higher expression of IL-6 *in situ*. Another study demonstrated that the levels of IL-6 in the umbilical cord were higher in placentas of prematures and that, in these cases, the frequency of prenatal sepsis and others occurrences were also higher [20]. Although the placental inflammation is not equivalent to intrauterine infection, there is a close relation between these two alterations [20, 21]. Our data reinforces this information demonstrating that the IL-6 variation in the fetal liver might follow the same changing pattern observed in the placenta. There was a negative correlation between the production of IL-6 and GA. This data is in accordance with studies on culture of LPS-stimulated amniotic cells, where the levels of IL-6 were inversely proportional to GA [21]. Interleukin-6 staining in liver sections might be associated with intrauterine infections and preterm delivery [22]. In accordance with these results, our data demonstrated higher IL-6 expression in preterm pregnancies. Here, CRP was strongly expressed in cases with AI, chronic stress, and in term children. The CRP is considered a good marker of AI, being more sensitive in late stages [23]. This data accords with our results, which have found an elevation in the expression of CRP in cases of chronic stress, specifically in cases of ascending infection, showing that exposure time is important so that the detection of CRP can be sufficient to diagnose intrauterine alterations. Moreover, there was a positive correlation between CRP expression and gestational age. As the CRP is synthesized by the liver, the variation in the CRP expression may be due to the functional immaturity of the fetal hepatocytes [24], demonstrating the premature child's nonresponse to aggressions. Interestingly, LGA and SGA children presented significantly higher expression of CRP. Higher maternal levels of CRP are associated with preeclampsia and growth-restricted baby [25]. In the same way, LGA children seem to go through oxidative stress, which may be responsible for fetal production of cytokines [26]. Therefore, in our cases, elevated levels of CRP in LGA and SGA children could be a consequence of oxidative stress and intrauterine growth restriction, respectively; both conditions reported to be related to excessive production of cytokines, including CRP [25, 26]. Since CRP does not cross the placental barrier, it may therefore be useful in diagnosing infections in newborns [27]. In the long term, these alterations would induce a higher production of CRP by the fetus, changing birth weight, as observed in this study.

In the present study, the expression of TNF-*α* was lower in cases with AI. A previous study has demonstrated that the amount of TNF-*α* decreases progressively 3 to 7 days after the treatment of sepsis whereas its values increase progressively in cases evolving into death. In this study, TNF-*α* was considered one of the best markers for the diagnosis of neonatal sepsis, and could be used to assess the effectiveness of the treatment and also the prognosis of the disease [28].

There was higher expression of TNF-*α* in the cases with PA. This data accords with other studies which demonstrate that hypoxia is the main cause of TNF-*α* increase [29]. As for the duration of stress, a study revealed that oxygen deprivation followed by reperfusion results in a significant increase of TNF-*α*. However, when the same procedure was repeated after a previous episode of ischemia and reperfusion, there was a protection against hepatic lesions, and also a not so important increase of TNF-*α* [30]. Thus, acute hypoxia may have been responsible for the high levels of TNF-*α* in cases with CM. There was a positive correlation between GA and the expression of TNF-*α*. Some studies show that the levels of TNF-*α* tend to decrease with the increase of the GA [31], whereas others suggest that the cytokines may be increased due to higher exposure to inflammatory stimuli in the uterus [32]. Our data indicates that GA influences the production of TNF-*α*, though the aggressions may have greater impact on its production in term and preterm infants, since different expressions between them were not observed.

There was a negative correlation between body weight and IL-6 but a positive correlation with CRP and TNF-*α* expression. It is believed that if the stress experienced is very intense, a release of great quantity of IL-6 may occur, causing loss of weight in the fetus due to the elevation of the catabolism [1]. The CRP is a marker of inflammation produced by the adipose tissue. Growth-restricted babies have, by definition, lower amounts of total fat mass compared with AGA babies, and it could suggest a more intense inflammatory state in the adipose tissue of the former [33]. In our data, one might infer that the positive correlation between CRP and body weight would represent a higher amount of fetal adipose tissue, or a more active adipose tissue, since many times the higher body weight in children with FIRS can be justified by edema caused by an inflammatory process in the face of aggressive agents [34]. TNF-*α* in inflammatory condition may be involved in alterations of the body weight through the association of the TNF-*α* with glucocorticoids as well as in promoting resistance to insulin [35]. Therefore, to complement the idea that the reduction of body weight is the first sign of intrauterine stress [36], we understand that the severe stress observed in our cases may have been responsible for the development of other alterations such as FIRS and also edema or lipid accumulation, resulting in the increase of body weight through different mechanisms [37]. Therefore, the weight as a fetal prognosis factor must be carefully evaluated, since the intensity of the stress factor may interfere directly in this parameter. We believe that future studies comparing the *in situ* versus plasmatic expression of markers of FIRS in children who evolved to death can elucidate mechanisms to predict the hepatic morphological status and the outcomes of FIRS in this organ.

## 5. Conclusions

In general, all FIRS markers were increased in cases with AI and were even higher in the other causes of death, especially IL-6 and CRP. Despite the diagnosis of FIRS being clinical and based on serum parameters, the findings in the current study allow the inference of FIRS diagnosis in the autopsied children, based on an *in situ* liver analysis of these markers. Further studies are needed to clarify if the *in situ* expression of such markers is an amplification of the body's response to FIRS or a reflex of the systemic fetal inflammation.

## Figures and Tables

**Table 1:** Perinatal stress classification according to perinatal cause of death.

Perinatal stress	Cause of death	Anatomopathologic diagnosis	Number of cases
Chronic	Perinatal anoxia	Meconium aspiration	2
		Fetal thrombotic vasculopathy	1
		Premature infants with anoxia	4
		Fetal erythroblastosis	2
		CM related to PA	4
		Hyaline membrane	2
		Anoxia—undetermined cause	6
	Ascending infection	Different degrees of chorioamnionitis	15

Acute	Congenital malformation	Genitourinary malformation	8
		Central nervous system malformation	4

**Table 2:** Analysis of the expression of IL-6, TNF-*α*, and CRP in the liver of perinatal autopsies.

Groups	*N* (%)	% IL-6	% CRP	% TNF-*α*
	48 (100)	Median (minimum–maximum)		
(A) Cause of death				
Perinatal anoxia	21 (43.75)	26.86 (2.41–88.18)^1,3^	3.07 (0.03–58.06)^5^	10.46 (0.01–54.89)^10,11^
Ascending infection	15 (31.25)	29.51 (1.26–78.75)^2,3^	5.22 (0.00–62.69)^4,5^	4.75 (0.02–57.44)^9,11^
Congenital malformation	12 (25.00)	32.91 (2.00–79.64)^1,2^	2.61 (0.02–46.83)^4^	9.30 (0.05–82.39)^9,10^
		*H* = 42.911; *P* ≤ 0.001	*H* = 28.920; *P* ≤ 0.001	*H* = 101.374; *P* ≤ 0.001
(B) Type of stress				
Chronic	36 (75.00)	27.85 (1.26–70.38)	3.63 (0.00–62.69)	8.35 (0.01–57.44)
Acute	12 (25.00)	32.91 (2.00–79.64)	2.61 (0.02–46.83)	9.30 (0.05–82.39)
		*U* = 1341457.000; *P* ≤ 0.001	*U* = 1212794.000; *P* ≤ 0.001	*U* = 1437612.000; *P* = 0.003
(C) Gestational age (GA)				
Term birth	11 (22.92)	25.97 (1.26–71.32)	8.45 (0.04–62.69)	7.39 (0.01–50.27)
Premature	37 (77.08)	29.82 (2.00–70.38)	2.85 (0.00–58.06)	9.22 (0.05–63.36)
		*U* = 1052865.000; *P* ≤ 0.001	*U* = 1449215.000; *P* ≤ 0.001	*U* = 1159934.500; *P* = 0.249
(D) Weight for GA				
SGA	6 (12.50)	27.42 (1.97–76.64)	4.79 (0.01–46.12)^7,8^	16.05 (0.02–82.39)^12,13^
LGA	12 (25.00)	29.76 (2.45–88.18)	5.89 (0.08–58.06)^6^	8.20 (0.04–55.02)^12^
AGA	30 (62.50)	29.40 (1.26–70.38)	2.67 (0.00–62.69)^6,7,8^	8.43 (0.01–57.44)^13^
		*H* = 0.0892; *P* = 0.956	*H* = 109.947; *P* ≤ 0.001	*H* = 30.065; *P* ≤ 0.001

^1 to 13 ^Dunn, *P*: <0.05. SGA: small for gestational age, LGA: large for gestational age, AGA: appropriate for gestational age.

**Table 3:** Perinatal weight distribution expected for GA among groups of causes of death in perinatal autopsies.

Groups *n* (%)		Gestational age (weeks) *X* ± SD	Perinatal weight (grams) *X* ± SD	EW (grams)	*P* ^♦^
Perinatal anoxia	21 (43.75)	32.4 ± 5.4	2110.48 ± 966.01	1488	<0.05
Ascending infection	15 (31.25)	31.3 ± 5.9	1828.73 ± 1035.47	1359	<0.05
Congenital malformation	12 (25.00)	33.5 ± 2.4	2225.00 ± 1149.84	1663	<0.05

Total	48 (100.00)				

^♦^“*t*” Student; *X*: mean; SD: standard deviation; EW: expected weight for population.

## References

[1] Madsen-Bouterse SA, Romero R, Tarca AL (2010). The transcriptome of the fetal inflammatory response syndrome. *The American Journal of Reproductive Immunology*.

[2] Bracci R (2000). Acute phase reaction in the fetus and newborn. *Acta Paediatrica*.

[3] Goepfert AR, Andrews WW, Carlo W (2004). Umbilical cord plasma interleukin-6 concentrations in preterm infants and risk of neonatal morbidity. *American Journal of Obstetrics and Gynecology*.

[4] David H (1985). The hepatocyte. Development, differentiation, and ageing. *Experimental Pathology, Supplement *.

[5] Arai H, Noguchi A, Goto R, Matsuda T, Nakajima H, Takahashi T (2010). Liver fibrosis in an extremely small infant for gestational age. *Tohoku Journal of Experimental Medicine*.

[6] Oliveira LF, da Silva Monteiro AP, Espindula AP (2012). Liver morphologic analysis in perinatal autopsies with intrauterine stress liver morphology in perinatal autopsies. *Fetal & Pediatric Pathology*.

[7] Amarilyo G, Oren A, Mimouni FB, Ochshorn Y, Deutsch V, Mandel D (2011). Increased cord serum inflammatory markers in small-for-gestational-age neonates. *Journal of Perinatology*.

[8] Campos DP, Silva MV, Machado JR, Castellano LR, Rodrigues V, Barata CHC (2010). Early-onset neonatal sepsis: cord blood cytokine levels at diagnosis and during treatment. *Jornal de Pediatria*.

[9] Hey EN, Lloyd DJ, Wigglesworth JS (1986). Classifying perinatal death: fetal and neonatal factors. *British Journal of Obstetrics and Gynaecology*.

[10] Corrêa RRM, Barrilari SEG, Guimarães CSO (2009). Expression of the melatonin receptor and tryptophan hydroxylase in placentas of the fetus with intra-uterine stress. *European Journal of Obstetrics Gynecology and Reproductive Biology*.

[11] Olegario JG, Silva MV, Machado JR (2013). Pulmonary innate immune response and melatonin receptors in the perinatal stress. *Clinical and Developmental Immunology*.

[12] Zago AFR, Paravidine LM, Siqueira LMS, Balbão LM, Reis MA, Castro EC (2000). Comparative study between foot length and other methods of gestational age determination in the newborn. *Pediatria Moderna*.

[13] FIGO (1976). International Federation of Gynaecology and Obstetrics definition of the midwife. *The Pakistan Nursing and Health Review*.

[14] Cartlidge PHT, Stewart JH (1995). Effect of changing the stillbirth definition on evaluation of perinatal mortality rates. *The Lancet*.

[15] Battaglia FC, Lubchenco LO (1967). A practical classification of newborn infants by weight and gestational age. *The Journal of Pediatrics*.

[16] Gruenwald P, Minh HN (1961). Evaluation of body and organ weights in perinatal pathology. II. Weight of body and placenta of surviving and of autopsied infants. *American Journal of Obstetrics and Gynecology*.

[17] Williams MA (1977). *Quantitative Methods in Biology*.

[18] Romero R, Savasan ZA, Chaiworapongsa T (2012). Hematologic profile of the fetus with systemic inflammatory response syndrome. *Journal of Perinatal Medicine*.

[19] Gonzalez BE, Mercado CK, Johnson L, Brodsky NL, Bhandari V (2003). Early markers of late-onset sepsis in premature neonates: clinical, hematological and cytokine profile. *Journal of Perinatal Medicine*.

[20] Kim CJ, Yoon BH, Park S-S, Kim MH, Chi JG (2001). Acute funisitis of preterm but not term placentas is associated with severe fetal inflammatory response. *Human Pathology*.

[21] Gomez R, Romero R, Ghezzi F, Bo Hyun Yoon BHY, Mazor M, Berry SM (1998). The fetal inflammatory response syndrome. *American Journal of Obstetrics and Gynecology*.

[22] Greksova K, Parrak V, Chovancova D (2009). Procalcitonin, neopterin and C-reactive protein in diagnostics of intrauterine infection and preterm delivery. *Bratislavské Lekárske Listy*.

[23] Døllner H, Vatten L, Austgulen R (2001). Early diagnostic markers for neonatal sepsis: comparing C-reactive protein, interleukin-6, soluble tumour necrosis factor receptors and soluble adhesion molecules. *Journal of Clinical Epidemiology*.

[24] Loukovaara M, Leinonen P, Teramo K, Alfthan H, Stenman U-H, Andersson S (2004). Fetal hypoxia is associated with elevated cord serum C-reactive protein levels in diabetic pregnancies. *Biology of the Neonate*.

[25] Tjoa ML, Van Vugt JMG, Go ATJJ, Blankenstein MA, Oudejans CBM, Van Wijk IJ (2003). Elevated C-reactive protein levels during first trimester of pregnancy are indicative of preeclampsia and intrauterine growth restriction. *Journal of Reproductive Immunology*.

[26] Lottenberg SA, Glezer A, Turatti LA (2007). Metabolic syndrome: identifying the risk factors. *Jornal de pediatria*.

[27] Rohde Nielsen F, Moller Bek K, Rasmussen PE, Qvist I, Tobiassen M (1990). C-reactive protein during normal pregnancy. *European Journal of Obstetrics Gynecology and Reproductive Biology*.

[28] Kocabaş E, Sarikçioğlu A, Aksaray N, Seydaoğlu G, Seyhun Y, Yaman A (2007). Role of procalcitonin, C-reactive protein, interleukin-6, interleukin-8 and tumor necrosis factor-*α* in the diagnosis of neonatal sepsis. *Turkish Journal of Pediatrics*.

[29] Kamiya A, Gonzalez FJ, Nakauchi H (2006). Identification and differentiation of hepatic stem cells during liver development. *Frontiers in Bioscience*.

[30] Teoh N, Leclercq I, Dela Pena A, Farrell G (2003). Low-dose TNF-*α* protects against hepatic ischemia-reperfusion injury in mice: implications for preconditioning. *Hepatology*.

[31] Romero R, Maymon E, Pacora P (2000). Further observations on the fetal inflammatory response syndrome: a potential homeostatic role for the soluble receptors of tumor necrosis factor *α*. *American Journal of Obstetrics and Gynecology*.

[32] Matoba N, Yu N, Mestan K (2009). Differential patterns of 27 cord blood immune biomarkers across gestational age. *Pediatrics*.

[33] Boutsikou T, Mastorakos G, Kyriakakou M (2010). Circulating levels of inflammatory markers in intrauterine growth restriction. *Mediators of Inflammation*.

[34] Romero R, Espinoza J, Gonçalves LF, Kusanovic JP, Friel L, Hassan S (2007). The role of inflammation and infection in preterm birth. *Seminars in Reproductive Medicine*.

[35] Rodrguez-Galn MC, Porporatto C, Sotomayor CE, Cano R, Cejas H, Correa SG (2010). Immunemetabolic balance in stressed rats during Candida albicans infection. *Stress*.

[36] Viengsakhone L, Yoshida Y, Harun-Or-Rashid M, Sakamoto J (2010). Factors affecting low birth weight at four central hospitals in vientiane, Lao PDR. *Nagoya Journal of Medical Science*.

[37] Djurhuus CB, Gravholt CH, Nielsen S (2002). Effects of cortisol on lipolysis and regional interstitial glycerol levels in humans. *The American Journal of Physiology—Endocrinology and Metabolism*.

